# SNAP23 Regulates Endothelial Exocytosis of von Willebrand Factor

**DOI:** 10.1371/journal.pone.0118737

**Published:** 2015-08-12

**Authors:** Qiuyu Zhu, Munekazu Yamakuchi, Charles J. Lowenstein

**Affiliations:** 1 Aab Cardiovascular Research Institute, Department of Medicine, University of Rochester Medical Center, Rochester, New York, United States of America; 2 Department of Pharmacology and Physiology, University of Rochester School of Medicine and Dentistry, Rochester, New York, United States of America; University of Miami School of Medicine, UNITED STATES

## Abstract

Endothelial exocytosis regulates vascular thrombosis and inflammation. The trafficking and release of endothelial vesicles is mediated by SNARE (Soluble NSF Attachment protein REceptors) molecules, but the exact identity of endothelial SNAREs has been unclear. Three SNARE molecules form a ternary complex, including isoforms of the syntaxin (STX), vesicle-associated membrane protein (VAMP), and synaptosomal-associated protein (SNAP) families. We now identify SNAP23 as the predominant endothelial SNAP isoform that mediates endothelial exocytosis of von Willebrand Factor (VWF). SNAP23 was localized to the plasma membrane. Knockdown of SNAP23 decreased endothelial exocytosis, suggesting it is important for endothelial exocytosis. SNAP23 interacted with the endothelial exocytic machinery, and formed complexes with other known endothelial SNARE molecules. Taken together, these data suggest that SNAP23 is a key component of the endothelial SNARE machinery that mediates endothelial exocytosis.

## Introduction

Endothelial cells (EC) maintain the integrity of the vasculature. In response to injury, EC undergo exocytosis, releasing numerous hemostatic and inflammatory mediators into the blood steam [1–3]. Weibel-Palade bodies (WPBs) are the secretory organelles specific to EC. Von Willebrand factor (VWF), a glycoprotein involved in hemostasis, is the major constituent released from WPBs. After its release, VWF initiates platelet adherence to the vessel wall, triggering a cascade of events that leads to thrombosis [2, 4–6]. Numerous studies have shown an association between plasma VWF levels and the risk of cardiovascular events: patients with increased VWF levels suffer a higher incidence of major adverse cardiac events [7, 8]. Enhanced understanding of the release of VWF may lead to novel treatments for vascular diseases such as atherosclerosis, stroke, myocardial infarction, and thrombosis.

Exocytosis is the process of cells releasing compounds by vesicle fusion with the plasma membrane [9–12]. The molecular machinery that regulates vesicle trafficking has been intensively studied in yeast and in neurons [13]. However, regulation of endothelial exocytosis is not well understood. Previous studies have identified some of the proteins that mediate endothelial exocytosis, including: NSF, vesicle-associated membrane protein (VAMP) 3, VAMP8, syntaxin 4 (STX4), myosin Va, MyRIP, Rab27a, Slp4-a, STXBP1, STXBP5, and RalGDS [2, 14–22]. Agonists such as thrombin trigger exocytosis by elevating intracellular Ca^2+^ levels, which initiate the final fusion of the vesicles with the plasma membrane [23]. Endogenous nitric oxide inhibits exocytosis by targeting NSF [14].

A superfamily of trans-membrane proteins called SNARE (Soluble NSF Attachment protein REceptors) play a central role in regulating specific membrane targeting and docking. One SNARE on a vesicle membrane (v-SNARE) binds to two counterpart SNARE on a target membrane (t-SNARE), forming a stable ternary complex that mediates granule exocytosis [12, 24]. The formation of a SNARE complex requires a four-helix bundle that brings the vesicle and target membrane in close apposition. VAMP isoforms and syntaxin isoforms each contribute one helix, and the other two helices are contributed by synaptosomal-associated protein (SNAP) isoforms [25]. For example, in neurons the specific interaction between VAMP2, syntaxin1A and SNAP25 regulates pre-synaptic vesicle priming and release. VAMP2 is localized on the membrane of pre-synaptic vesicles, and syntaxin 1a and SNAP25 are localized to the neuronal presynpatic membrane.

The precise identity of the three SNARE molecules that regulate endothelial exocytosis are not completely clear. Potential candidates include VAMP isoforms (VAMP3 and VAMP8), STX4, and SNAP isoforms [14, 15, 26–28]. However, SNAP25 is present almost exclusively in the brain. In endothelial cells, SNAP25 is not detectable, suggesting that a homolog of SNAP25 mediates endothelial SNARE complex [2, 14, 29].

SNAP23, a ubiquitously-expressed homolog of SNAP25, shares 59% identical to SNAP25. SNAP23 can regulate exocytosis in several distinct cell types. SNAP23 is localized to the plasma membrane in adipocytes and interacts with multiple syntaxin isoforms (syntaxin 2, 3, 4, and 5) [30]. SNAP23 regulates GLUT4 translocation, neuroendocrine cell exocytosis, and mast cell degranulation [30–34], suggesting SNAP23 appears to fulfill the function of SNAP25 in non-neuronal tissues in forming SNARE complex. SNAP23 has been found in human endothelial cells [14, 15]. SNAP23 interacts with Cav-1 and plays an important role in endothelial caveolae transcytosis [27]. However, studies of the role of SNAP23 in endothelial exocytosis are limited: partial knockdown of SNAP23 led to a non-significant decrease in exocytosis [15]. Therefore, in an effort to resolve the ambiguities surrounding the function of SNAP23 in endothelial exocytosis, we used enhanced EC culture methods and an exocytosis assay to explore the role of SNAP23 in endothelial exocytosis.

## Materials and Methods

### Materials and Reagents

Histamine, human thrombin, and calcium ionophore A23187 were purchased from Sigma-Aldrich. Rapamycin was purchased from LC Laboratories. Mouse monoclonal antibody to VWF, rabbit polyclonal antibodies to SNAP23 and STX4 were purchased from Abcam. Mouse monoclonal antibody to STX4 was purchased from BD Transduction Laboratories. Polyclonal antibodies to VAMP3, GAPDH, and β-actin was purchased from Santa Cruz Biotechnology. Goat polyclonal antibody to VAMP8, and sheep polyclonal antibodies to SNAP25 and SNAP29 were purchased from R&D Systems. Affinity purified polyclonal antibodies to SNAP47 was from Synaptic Systems. Mouse polyclonal SNAP91 antibody was from Sigma Life Science. Rabbit monoclonal antibody to LC3B (D11) was from Cell Signaling Technology.

### Cell Culture

Human umbilical vein endothelial cells (HUVEC), human dermal microvascular endothelial cells (HDMVEC) and human aortic endothelial cells (HAEC) were purchased from Lifeline Cell Technology and cultured in VascuLife EnGS Medium (Lifeline Cell Technology) containing cell-specific growth supplement. Cells from passages 2–6 were used for experiments. Human brain microvascular endothelial cells (HBMEC) was purchased from Lonza and cultured in VascuLife Basal Medium (Lifeline Cell Technology) supplemented with growth factors for human microvascular endothelial cells. Cells were maintained at 37°C and 5% CO_2_ with humidity. Cell confluence was visually determined when cells were in contact and the entire culture surface had no visible space among individual cells for at least 48 h. For autophagy induction, cells were starved with HBSS for 1 h at 37°C, or 250 nM rapamycin in complete medium for 12 h at 37°C.

### Microarray

Total RNA was extracted from HUVEC from six different donors by RNeasy mini kit (Qiagen) according to the manufacturer’s protocol. The gene expression profiling was performed using Affymetrix GeneChip at the Genomics Research Center at University of Rochester.

### RT-qPCR

Mouse tissues were harvested from healthy 8-week-old male C57BL/6 mice, after euthanasia with CO_2_ inhalation and cervical dislocation. The procedures and usage of mice were approved by the Division of Laboratory Animal Medicine at the University of Rochester Medical Center. HUVEC and murine tissue mRNA was isolated by TRIZOL reagent (Invitrogen) and purified by LiCl. Reverse transcription was performed using High-Capacity cDNA Reverse Transcription kit (Applied Biosystems). Quantification of SNAP homologs was performed by two-step RT-qPCR. Triplicates per gene per sample were performed on the same plate. For HUVEC, real-time PCR was performed using SYBR Green PCR Master Mix (Applied Biosystems) in 15 μl volume with 10 ng cDNA and 250 nM primers for 40 cycles followed by melt curve analysis on an iCycler thermal cycler with MyiQ detection system (Bio-Rad) (Table 1). *SNAP* homolog expression was quantified by ΔΔCt method using 4 reference genes: *B2M*, *GAPDH*, *HRPT1*, and *YWHAZ*, and expressed as percentage relative to the amount of *SNAP23*. For murine *Snap23* quantification, Taqman gene expression assay was performed with TaqMan Universal PCR Master Mix (Applied Biosystems) following the manufacture’s protocol, and murine *Actb* was used as reference gene for quantification. Taqman probes were purchased from Applied Biosystems. All RT-qPCR products were separated on agarose gels to confirm absence of primer-dimer and nonspecific products.

**Table 1:** Primers used for SYBR Green RT-qPCR.

**Gene**	**RefSeq**	**Forward (5'>3')**	**Reverse (5'>3')**	**Product length (bp)**
***SNAP23***	NM_003825.3	ATGAGTCTCTGGAAAGTACGAGG	CCACAGCATTTGTTGAGTTCTG	190
***SNAP25***	NM_003081.3	TGTTGGATGAACAAGGAGAACAA	CCGTCCTGATTATTGCCCCA	187
***SNAP29***	NM_004782.3	CCTGAACAGAATGGCACCCT	TGGGGACAGGGTCTGTATCA	139
***SNAP47***	NM_053052.3	TGGAGGTGGCGGACAGATT	AGGGTTCACAACTGGTCATGG	129
***SNAP91***	NM_001242792.1	AGCCGGTCATGTTTGCACA	AGATCCGCTAATGGGTCCTTT	139

### Transcriptional profile by ENCODE

The Feb 2009 GRCh37/hg19 Assembly was searched for transcription levels of SNAP homologs. Transcriptional profiles were visualized in UCSC Genome Browser with a customized ENCODE track for HUVEC.

### RNA interference

HUVEC and HDMVEC were plated on collagen I coated 96-well plates until reaching 80–90% confluence [35]. Transfection was performed using Lipofectin reagent (Invitrogen) and Opti-MEM I Reduced Serum Media (Invitrogen) for 5 hours with 20 nM Stealth RNAi siRNA (Ambion) against SNAP23, SNAP25, SNAP29, SNAP27, and SNAP91. After 72 hours post-transfection cells were stimulated for exocytosis or directly lysed for SDS-PAGE.

### VWF Exocytosis Assay

VWF exocytosis assay was performed as described [14, 21, 29, 35]. Briefly, we replaced medium of confluent cells with either pre-warmed serum-free medium (resting), or serum-free medium containing 10 μM histamine, or 1 U/ml thrombin, or 10 μM A23187 (stimulation). Cells were maintained at 37°C, 5% CO_2_ with agonists in a vibration-free incubator. After 30 min, the medium was collected and VWF content measured with IMUBIND VWF ELISA (Sekisui Diagnostics). Alternatively, cells were lysed immediately after medium removal without stimulation, with total protein normalized to measure total intracellular VWF contents.

### Cell Fractionation

Cytosolic and membrane fractions of HUVEC were prepared using a cell fractionation kit (Cell Signaling Technology) with 5×10^6^ of cells for both sub-confluent and confluent conditions. For sucrose density gradient ultracentrifugation, HUVEC was lysed on ice using 1 ml M-PER mammalian protein extraction reagent (Thermo Scientific) supplemented with EDTA-free protease inhibitor cocktail tablet (Roche Diagnostics) and spun to pellet the cell debris. The supernatant was loaded on top of a sucrose gradient consists of 1 ml 5% sucrose, 6 ml 30% sucrose, and 3 ml 40% sucrose in a 14 ml PET thin-walled tube (Thermo Scientific). Ultracentrifugation was performed with SureSpin 630/17 swinging-bucket rotors (Sorvall) at 166,880 g for 20 hours at 4°C, after which 18 equal-volume aliquots were collected from top to bottom and analyzed by Western blot.

### Western Blot

Cells lysate was mixed directly with Laemmli sample buffer (Bio-Rad). The samples were boiled and resolved by SDS-PAGE as described [21].

### Co-Immunoprecipitation

HUVEC were treated with agonists and lysed on ice using M-PER mammalian protein extraction reagent containing protease inhibitor and spun 15min twice at 161, 000 g to remove insoluble contents, and pre-cleared with 25 μl protein A/G PLUS-agarose (Santa Cruz Biotechnology) for 1.5 h at 4°C. 25 μl protein A/G PLUS-agarose was incubated with 2 μg antibody or isotype IgG overnight at 4°C and pelleted to mix with the pre-cleared supernatant and incubated overnight at 4°C. The beads were washed 6 times with cold PBS and eluted with Laemmli sample buffer at 95°C followed by SDS-PAGE.

### Confocal Microscopy

HUVEC were cultured on collagen-coated glass-bottom dishes (MatTek), fixed with 4% paraformaldehyde in PBS, permeabilized with 0.15% Triton X-100 for 20 minutes, and blocked with 5% donkey serum for 1 hour at room temperature. Cells were immunostained with primary antibodies at 1:330–1:1000 dilution range overnight at 4°C, washed, and incubated for 1 h at room temperature with DAPI and secondary fluorescent labeling antibodies (all at 1:2000 dilution), including Alexa Fluor 488 goat anti-mouse IgG, Alexa Fluor 546 goat anti-mouse IgG, Alexa Fluor 488 goat anti-rabbit IgG, Alexa Fluor 594 goat anti-rabbit IgG, Alexa Fluor 680 donkey anti-sheep IgG (Molecular Probes), Cy3-AffiniPure Bovine Anti-Goat IgG (H+L) (Jackson ImmunoResearch Laboratories). Cells were imaged on an IX81 inverted confocal microscope with an FV1000 camera (Olympus) using sequential line scanning. FV10-ASW 3.0 (Olympus) software was used for image capture and analysis.

### Statistics

Results were expressed as mean ± SD. Significance between mean values was determined by the two-tailed Student's t-test for comparison between two groups, with P < 0.05 defined as statistically significant. One-way ANOVA with Tukey multiple comparisons test was performed for comparison among three or more groups. Responses affected by two factors were compared by two-way Tukey-corrected ANOVA. For ANOVA post tests, multiplicity adjusted P < 0.05 was considered as significant.

## Results

### SNAP23 is Expressed in Human Endothelial Cells and Murine Tissues

**Fig 1:**
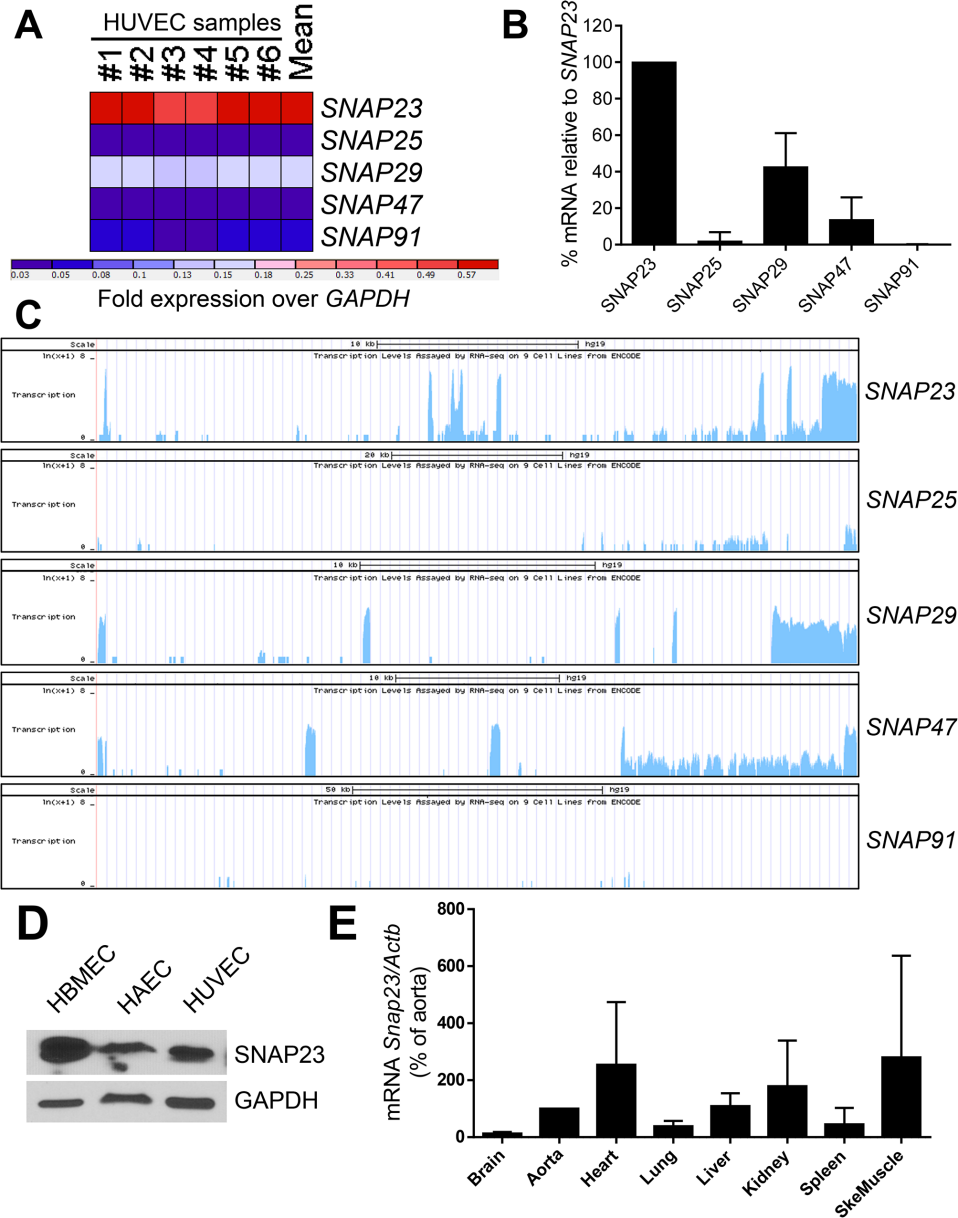
SNAP23 is expressed in human endothelial cells and murine tissues. (A) Heat map of the gene expression values for SNAP homologs in 6 samples of human umbilical vein endothelial cells (HUVEC) by microarray. Relative expression values were normalized to that of *GAPDH*. (B) Relative expression of SNAP homologs in 3 individual donors of HUVEC as assayed by RT-qPCR. Expression was normalized as percentage of *SNAP23* expression. Primers listed in Table 1. (C) ENCODE data on the UCSC genome browser depicting the expression of HUVEC SNAP homologs as assayed by RNA-seq. (D) SNAP23 is expressed in human brain microvascular endothelial cells (HBMEC), human aortic endothelial cells (HAEC), and HUVEC as measured by Western blot. (E) SNAP23 is expressed in multiple murine tissues as measured by qRT-PCR (n = 3 ± SD).

In order to define the SNAP homologs that regulates release of endothelial granules, we first searched for endothelial expression of SNAP homologs. Using microarray hybridization techniques, we found that HUVEC express mRNAs for *SNAP23*, *SNAP25*, *SNAP29*, *SNAP47*, and *SNAP91* (Fig 1A). Abundance of these mRNA homologs were confirmed by RT-qPCR (Fig 1B). Transcription of these SNAP homologs in HUVEC was further confirmed using ENCODE RNA-Seq data (Fig 1C). The SNAP homolog expressed at highest levels in endothelial cells is *SNAP23* (Fig 1A and 1B). SNAP29 and SNAP47 homologs were detected at lower levels by qRT-PCR (Fig 1B) but not by immunoblot (S1 Fig).

We next characterized the expression of SNAP23 in human endothelial cells and murine tissues. We found SNAP23 is expressed in different human endothelial cell types by Western blot, including human brain microvascular endothelial cells (HBMEC), human aortic endothelial cells (HAEC), and HUVEC (Fig 1D). Expression of SNAP29 and SNAP47 was minimal in HUVEC by Western blot (S1 Fig). *Snap23* mRNA is expressed in murine heart, kidney, skeletal muscle, and other tissues (Fig 1E). Our results extend previous studies showing *SNAP23* mRNA is ubiquitously expressed, although its tissue abundance varies between mouse and human [36, 37].

### SNAP23 is the only SNAP Homolog Localized on Endothelial Cell Membranes

**Fig 2:**
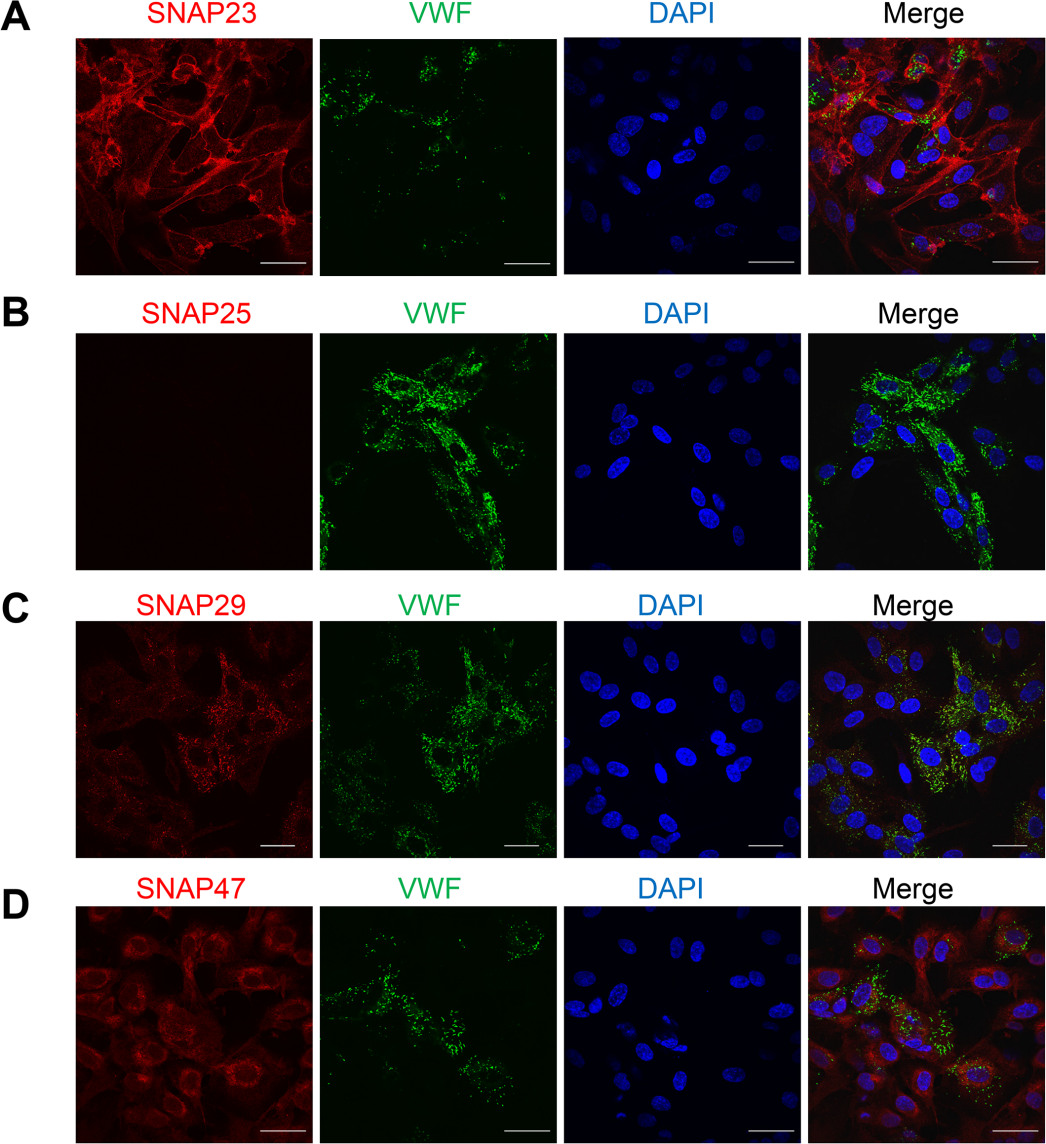
Subcellular localization of SNAP homologs in endothelial cells. Confocal microscopy was used to define the location of SNAP homologs (red) including SNAP23 (A), SNAP25 (B), SNAP29 (C), SNAP47 (D), VWF (green), and DNA (blue), and the images were merged. Only SNAP23 is localized to the plasma membrane.

We expected that the SNAP homolog that regulates endothelial granule fusion for exocytosis is located on the plasma membrane. We used confocal microscopy to compare the subcellular localization of SNAP homologs detected by qRT-PCR in endothelial cells. We focused on the location of SNAP homologs relative to the endothelial WPBs that contain VWF as well as other pro-thrombotic and pro-inflammatory compounds. SNAP23 is primarily localized to the plasma membrane (Fig 2A). VWF is the major component of endothelial granules that is released by endothelial exocytosis [38], which is localized to the typical cigar-shaped WPB granules (Fig 2). There is no significant overlap between SNAP23 and VWF staining (Fig 2A). SNAP25 is expressed in low levels measured by qRT-PCR (Fig 1B) and is not visible in endothelial cells by confocal microscopy (Fig 2B). SNAP29 is located in a particulate compartment and partially co-localizes with VWF, but SNAP29 is not located on the plasma membrane (Fig 2C). SNAP47 is located in a perinuclear distribution, with no over-lapping with VWF (Fig 2D). SNAP91 was not detected in endothelial cells (Fig 1B) and we did not measure its location by confocal microscopy. In conclusion, SNAP23 is the only SNAP homolog localized to the endothelial plasma membrane.

Since SNAP29 is partially co-localized with VWF containing granules (Fig 2C), we further explored the location of SNAP29 in endothelial cells. Prior studies have shown that SNAP29 mediates membrane fusion between intracellular organelles and the lysosome [39–46]. Additional studies have shown that autophagy in endothelial cells regulates VWF synthesis, maturation, and secretion [47]. Our discovery that SNAP29 partially co-localizes with VWF prompted us to further explore its role in endothelial autophagy. We found that SNAP29 partially co-localizes with VWF in resting endothelial cells (S2A Fig), and also co-localizes with autophagosome marker LC3B (S2B Fig). To further explore the role of SNAP29 in autophagy, we induced autophagy by starvation or rapamycin. Autophagy increased the intensity of autophagosomes marked by LC3B, and increased the co-localization of SNAP29 with VWF and LC3B (S3 Fig). Autophagy also leads to a change in the morphology of VWF granules from rods to spheres (S3 Fig) [47]. Taken together these results suggest that SNAP29 may play a role in autophagy in endothelial cells, possibly mediating fusion of endothelial granules with the autophagosome. However, our data show that SNAP29 is not located on the plasma membrane, and thus cannot function as a target SNARE that mediates endothelial granule fusion with the plasma membrane.

### SNAP23 is the only SNAP Homolog that Regulates Endothelial Exocytosis

**Fig 3:**
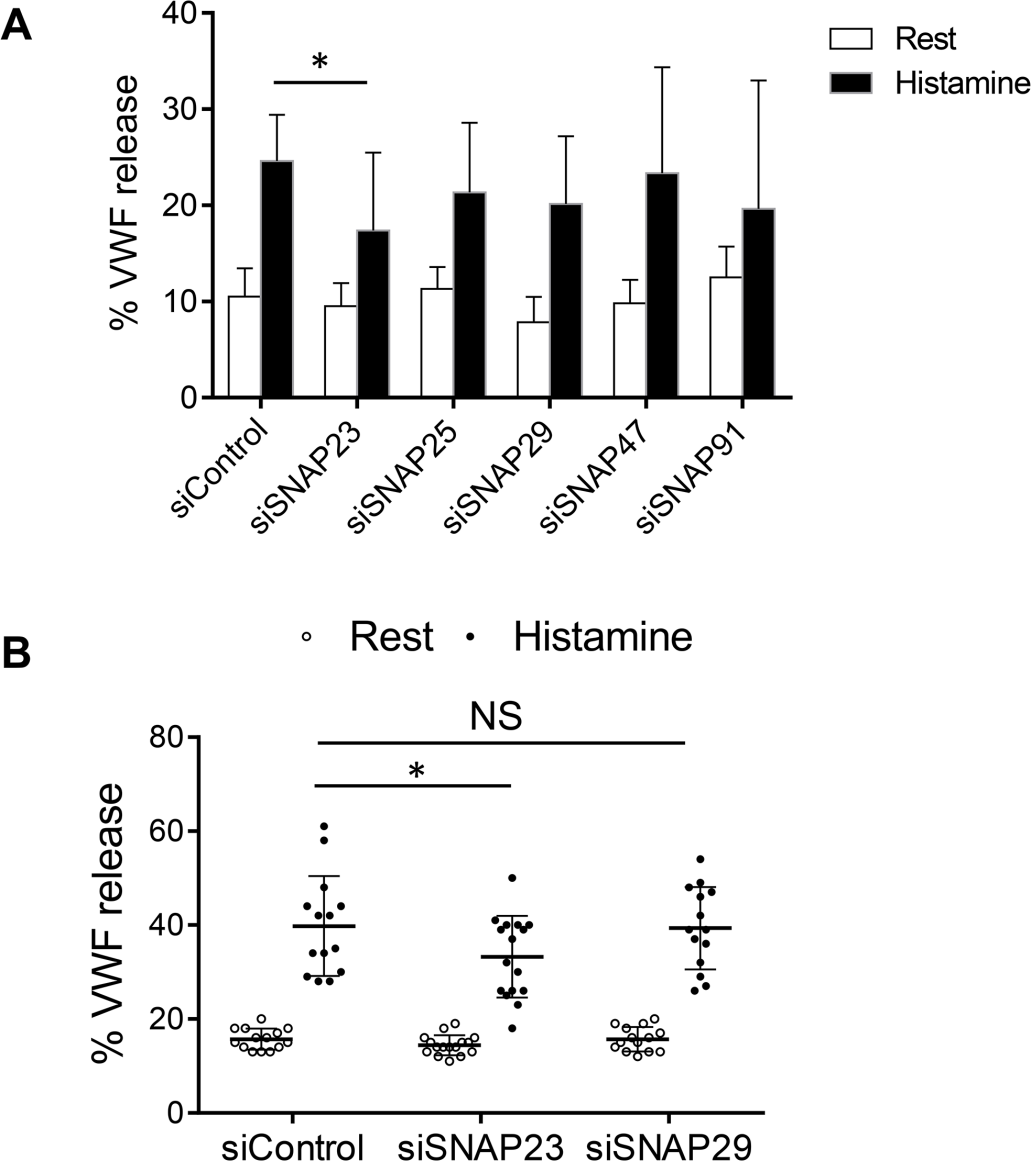
SNAP23 but not other SNAP homologs regulate VWF exocytosis. (A) The effect of siRNA targeting individual SNAP homologs. HUVEC were transfected with siRNA against SNAP homologs or control siRNA, stimulated with media or histamine, and VWF release was measured by an ELISA. Knockdown of SNAP23 decrease stimulated VWF release, but knockdown of other SNAP homologs has no effect on VWF release (n = 4–6 ± S.D.). (B) Comparison of the role of SNAP23 and SNAP29 in mediating exocytosis. The effect of SNAP23 and SNAP29 in endothelial exocytosis after RNAi was measured as above. Knockdown of SNAP23 decreased exocytosis, but knockdown of SNAP29 had no effect (n = 14–16 ± S.D.).

Which SNAP homolog regulates endothelial exocytosis? To answer this question, we first knocked down endothelial expression of each SNAP homolog with siRNA, then stimulated the endothelial cells with control or histamine and measured the release of VWF into the media. Knockdown of SNAP23 decreases exocytosis of VWF compared to knockdown with a control siRNA (Fig 3A). Knockdown of other SNAP homologs had minimal effect on histamine induced VWF release (Fig 3A).

Since SNAP29 partially co-localizes with VWF containing granules, we repeated our knockdown experiments of SNAP23 or SNAP29 only. We again found that knockdown of SNAP23 decreases VWF exocytosis but knockdown of SNAP29 has no effect (Fig 3B).

### SNAP23 Regulates Endothelial Exocytosis

**Fig 4:**
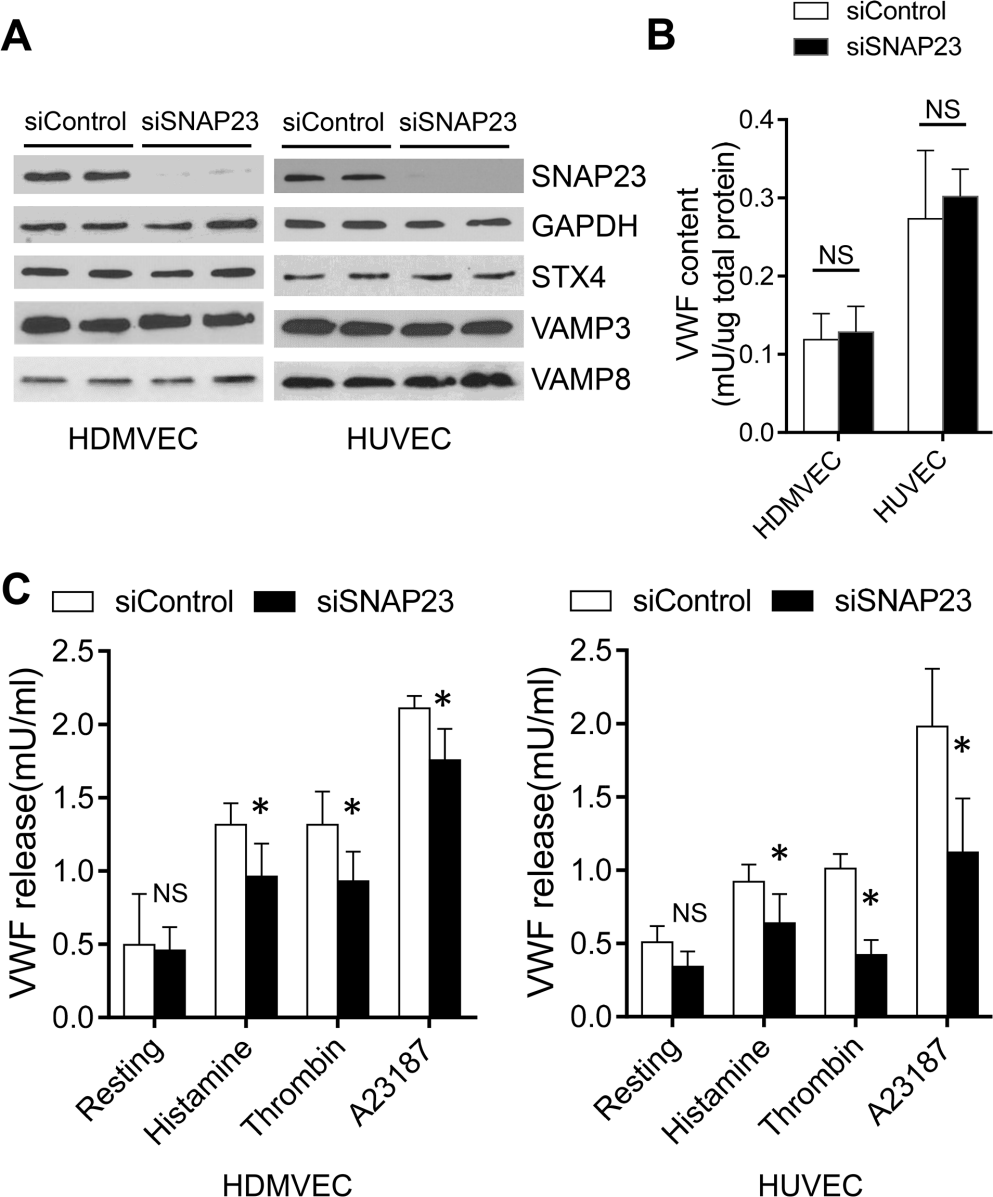
SNAP23 is important for endothelial exocytosis. (A) siRNA against SNAP23 knocks down SNAP23 protein levels as measured by Western blot. The siRNA against SNAP23 has no effect on the expression of other SNARE proteins including STX4, VAMP3, and VAMP8. GAPDH was used as loading control. (B) SNAP23 knockdown does not affect VWF expression in HDMVEC or HUVEC. Total VWF content was measured in total cell lysate by an ELISA in control siRNA and siSNAP23 treated cells (n = 6; NS, non-significant). (C) SNAP23 knockdown decreases endothelial exocytosis. HDMVEC and HUVEC were treated with siControl or siSNAP23, stimulated with serum-free medium only (resting), or 10 μM histamine, or 1 U/ml thrombin, or 10 μM Ca^2+^ ionophore A23187 for 30 min; and then VWF released into the media was measured by an ELISA (n = 4–7; * P < 0.05 vs. siControl; NS, non-significant vs. siControl). Data are represented as mean ± SD.

We next explored the role of SNAP23 in endothelial exocytosis. We knocked down the expression of endogenous SNAP23 in human dermal microvascular endothelial cells (HDMVEC) and HUVEC, stimulated the cells to trigger exocytosis, and then measured the amount of VWF released into the media by an ELISA. Expression of SNAP23 was significantly reduced by siRNA (Fig 4A). The expression of other SNARE proteins in endothelial cells were not affected by siRNA against SNAP23, including STX4, VAMP3, and VAMP8 (Fig 4A). The total intracellular VWF content was also unaffected by SNAP23 knockdown (Fig 4B). We found that knockdown of SNAP23 significantly reduced VWF exocytosis induced by the physiological agonists histamine and thrombin, as well as by the Ca^2+^ ionophore A23187 (Fig 4C). Knockdown of SNAP23 decreases exocytosis between by approximately 29% in HDMVEC to 58% in HUVEC (Fig 4C). Taken together, these results suggest SNAP23 regulates Ca^2+^-dependent endothelial exocytosis.

### Cell confluency affects membrane distribution of SNAP23

**Fig 5:**
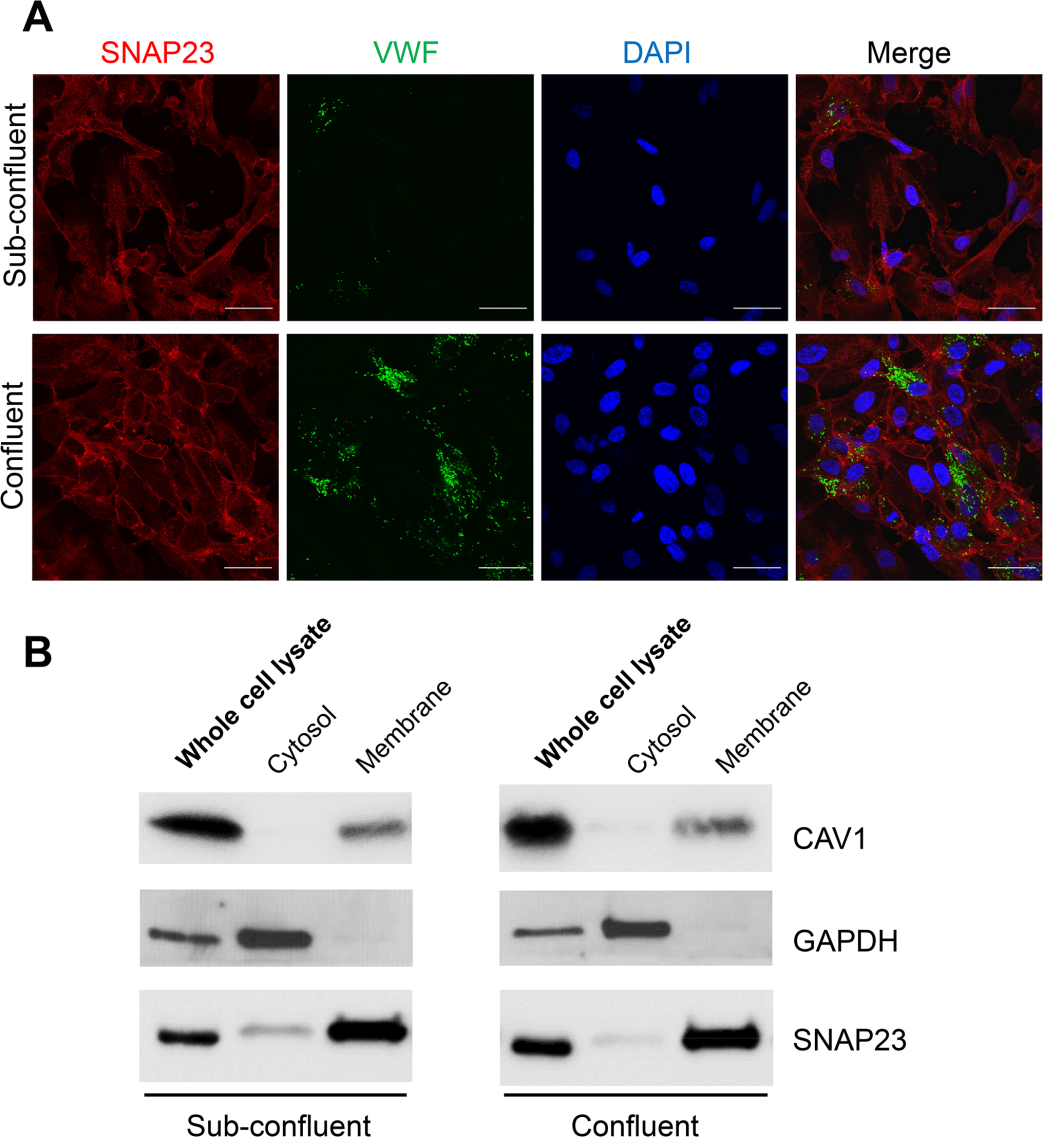
Subcellular localization of SNAP23 in endothelial cells. (A) Subcellular localization of SNAP23 in sub-confluent (upper panel) and confluent (lower panel) endothelial cells. Immunofluorescent staining was performed on HUVEC with antibodies against SNAP23 (red) and VWF (green), DNA was stained with DAPI (blue), and the cells were imaged by confocal microscopy (objective 60× oil, scale bar = 40 μm, confocal z resolution = 0.32 μm). (B) Western blot analysis of cell fractions from sub-confluent and confluent HUVEC using markers for membrane (caveolin-1 or CAV1) and cytosol (GAPDH). These fractions were also probed for SNAP23. Whole cell lysates were used for total protein. SNAP23 expression is decreased from cytosol fraction when cells are confluent.

Prior studies have shown that SNAP23 is partially localized to the plasma membrane and the cytoplasm [48–50]. As we studied the location of SNAP23 by confocal microscopy, we noticed that confluency affected SNAP23 subcellular location. Fully confluent cells have prominent cell membrane staining of SNAP23 and less cytoplasmic SNAP23, whereas subconfluent cells showed more SNAP23 in the cortical region and the cytoplasm (Fig 5A). To confirm this observation, we cultured HUVEC at sub-confluent and confluent conditions, isolated cytosol and membrane fractions, and immunoblotted fractions for SNAP23. SNAP23 is mostly found on the membrane fraction of subconfluent cells and confluent cells (Fig 5B). More SNAP23 protein was detected in the cytosol of sub-confluent cells than of confluent cells (Fig 5B). Taken together, these data suggest SNAP23 is primarily localized on the plasma membrane in endothelial cells, and its cytosolic distribution depends in part on cell confluence.

### SNAP23 Interacts with Endothelial Exocytic Machinery

**Fig 6:**
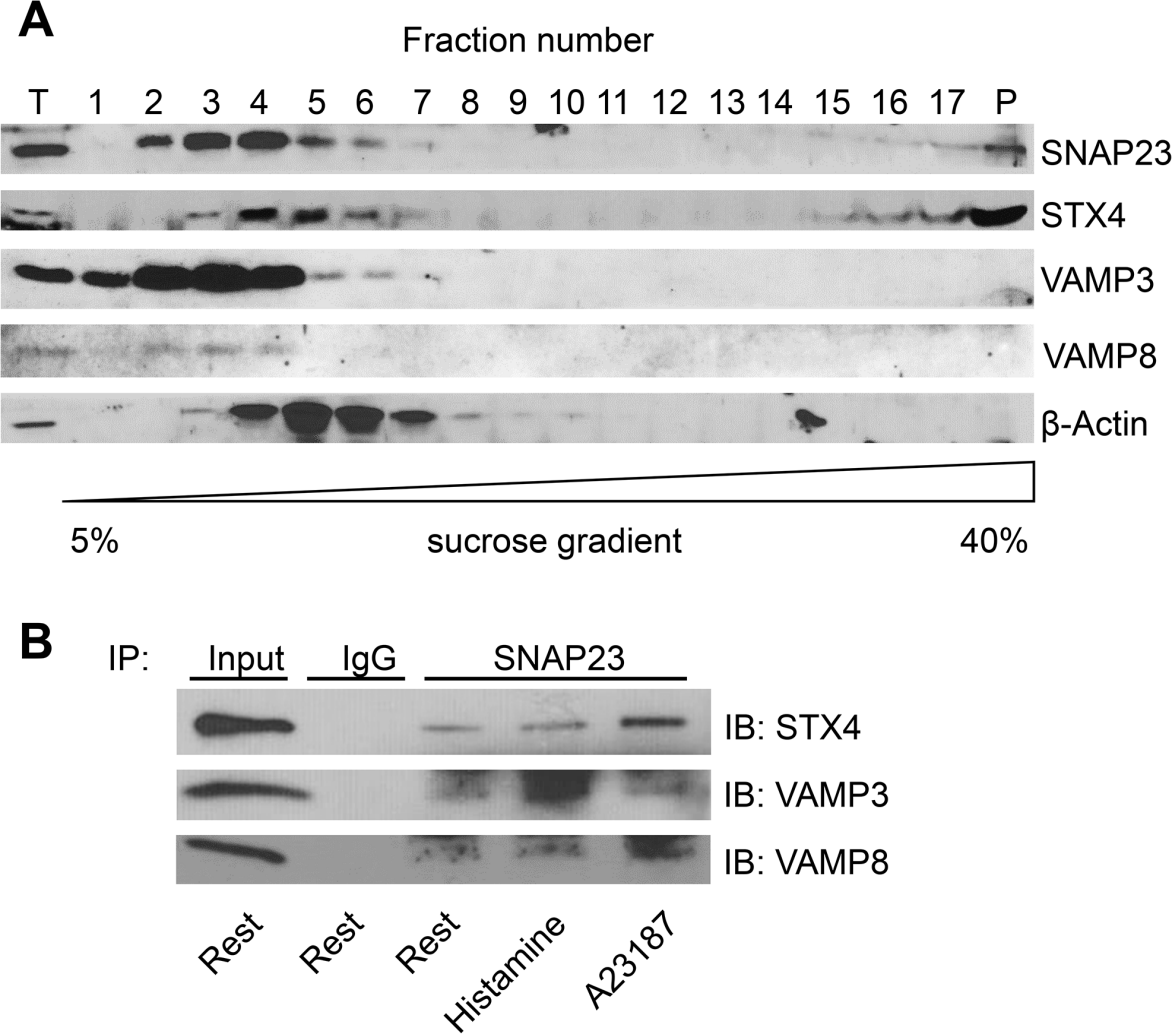
SNAP23 interacts with endothelial exocytic machinery. (A) SNAP23 co-sediments with STX4, VAMP3 and VAMP8 as analyzed by sucrose density gradient fractionation. HUVEC lysates were ultracentrifuged through a 5%–40% discontinuous sucrose gradient, and then the gradient was aliquoted into 17 fractions and analyzed by SDS-PAGE (T, total proteins in the lysate; P, pellet after fractionation). β-actin was used as control for fraction separation. Representative of 3 separate experiments. (B) SNAP23 co-precipitates with STX4, VAMP3 and VAMP8. HUVEC were stimulated with serum-free medium only (Rest), or 10 μM histamine, or 10 μM Ca^2+^ ionophore A23187 for 30 min; and cell lysates were immunoprecipitated with antibody to SNAP23 or isotype IgG. The precipitants were probed with antibody to STX4, VAMP3 and VAMP8. Input represents 5% total protein. Representative of 3 similar experiments. SNAP23 co-precipitates with STX4, VAMP3, and VAMP8.

Since SNAP23 is important for endothelial exocytosis, we next searched for a link between SNAP23 and the endothelial exocytic machinery. In order to search for the interaction partners of SNAP23, we first performed sucrose density gradient fractionation. We separated HUVEC lysates through a 5%–30%–40% discontinuous sucrose gradient, and probed 17 fractions for SNARE proteins involved in endothelial exocytosis. SNAP23 co-sediments with STX4 in fractions 3 to 7 and 15 to P (Fig 6A). SNAP23 partially co-sediments with the endothelial SNARE molecules VAMP3 and VAMP8 (Fig 6A). The sucrose density gradient fractionation provided indirect evidence that SNAP23 may interact with endothelial SNARE molecules. To confirm their interaction in a complex, we immunoprecipitated HUVEC lysates with antibody to SNAP23 or isotype matched IgG, and then probed precipitants for SNARE proteins. STX4, VAMP3, and VAMP8 were all detectable in the precipitant, in resting and stimulated cells (Fig 6B). To complement these data, we repeated our localization experiments. We used confocal microscopy to measure the co-localization of endothelial SNARES in resting and stimulated cells. SNAP23 co-localizes with STX4 in resting and stimulated cells (Pearson’s correlation coefficient of 0.47 ± 0.02 in resting cells and 0.48 ± 0.05 in stimulated cells) (S4 Fig and S5 Fig). However, SNAP23 has less co-localization with VAMP3 (Pearson’s correlation coefficient of 0.23 ± 0.02 in resting cells and 0.28 ± 0.04 in stimulated cells) and with VAMP8 (Pearson’s correlation coefficient of 0.16 ± 0.04 in resting cells and 0.15 ± 0.03 in stimulated cells) (S4 Fig and S5 Fig). Taken together, these data suggest SNAP23 interacts in a complex with components of the endothelial exocytic machinery containing STX4, VAMP3, and VAMP8, both under resting and stimulated conditions.

## Discussion

The identity of the synaptosomal-associated protein which functions as a t-SNARE in endothelial cell exocytosis is unknown. In this study, we found that SNAP23 is the most highly expressed SNAP isoform in different types of human endothelial cells; SNAP23 is localized to the endothelial cell membrane; SNAP23 forms complexes with other endothelial SNARE molecules; and most importantly, deficiency of SNAP23, but not the other endothelial SNAP homologs, impairs endothelial exocytosis. These results collectively suggest that SNAP23 plays a critical role in regulating endothelial cell exocytosis.

Our work and the studies of others show that human endothelial cells express a distinctive subset of SNARE molecules [2, 14, 15]. Endothelial cells express VAMP3 and VAMP8 of the v-SNARE family, STX4 of the syntaxin family, and several SNAP isoforms including SNAP23. These results demonstrate that endothelial cells express specific family members of the exocytic machinery also found in neurons and yeast. We also found that a subset of endothelial SNAREs interact with each other: endothelial cells contain a SNARE complexes consisting of SNAP23, STX4, and VAMP3 or VAMP8 (Fig 6B). This SNARE complex corresponds to SNARE complexes found in neurons, composed of SNAP25, STX1a, and VAMP2 [11, 12]. We also found that SNAP23 co-sediments with STX4 and VAMP3 and VAMP8 (Fig 6A). Our work supports the studies of others that show SNAP23 and STX4 form clusters in endothelial cells, and the results of others showing SNAP25 and STX4 form clusters in neuroendocrine cells [51, 52].

In resting cells, SNAP23 interacts with STX4, VAMP3, and VAMP8 (Fig 6). Stimulation of endothelial cells with histamine or calcium ionophore increases the interaction of SNAP23 with STX4, VAMP3, and VAMP8 (Fig 6B). These data support the idea that SNAP23 functions as one component of the ternary SNARE complex in endothelial cells. Our work partially contrasts with the work of others, who show that SNAP23 is localized to plasma membrane but has little effect on endothelial exocytosis [15]. One possible explanation for this discrepancy is that knockdown of SNAP23 expression was incomplete in other studies.

Among the members of the SNAP family, only two homologs have been regarded as critical components in exocytosis, SNAP25 and SNAP23. SNAP25 is expressed in neuronal and neuroendocrine tissues, whereas SNAP23 is ubiquitously expressed in non-neuronal cells [36]. Endothelial cells express lower levels of other SNAP isoforms (Fig 1A–1C).

We show that SNAP23 is localized to endothelial plasma membranes (Figs 2 and 5). Our data suggest SNAP23 clearly plays a role similar to SNAP25 in neurons, serving as a t-SNARE. However, a minor fraction of SNAP23 was also found in the cytosol (Fig 5), similar to previous studies [27]. It has been previously confirmed that the plasma membrane localization of SNAP family proteins depends on the palmitoylation of a cysteine-rich domain [53]. It is plausible that a sub-fraction of SNAP23 proteins is detectable in the cytosol before palmitoylation occurs. Cytosolic levels of SNAP23 are decreased in confluent cells (Fig 5B), suggesting that palmitoylation of SNAP23 may be dynamically regulated and is not constitutive. The function of SNAP23 in the cytosol is unclear: SNAP23 could be trafficking from the ER to Golgi to plasma membrane, or it could be serving another undefined role.

SNAP29 is the second most abundant SNAP homolog in human endothelial cells. SNAP29 was first discovered in 1998 from a human brain cDNA library [54], later found as a promiscuous syntaxin-binding SNARE in various membrane structures [55–61]. SNAP29 protein shares limited (17%) identity to SNAP23 and SNAP25. One unique feature of SNAP29 is it does not have a hydrophobic transmembrane domain or the palmitoylated cysteine residues in SNAP23 or SNAP25 for membrane anchoring. Instead, SNAP29 localizes to many intracellular membrane compartments via its interaction with multiple syntaxins and VAMPs which are already on the membrane. SNAP29 participates in membrane fusion between various intracellular compartments [62], including post-Golgi vesicle fusion with endosomes [57, 63, 64], autophagosome fusion with a lysosome [39, 65, 66], post-fusion SNARE disassembly or turnover [56, 60], endocytic recycling [67], and phagocytosis [61].

We found SNAP29 is linked to autophagy in endothelial cells. First, SNAP29 is partially localized to VWF and the autophagosome marker LC3B (S1 Fig and S2 Fig). Furthermore, induction of autophagy by starvation or rapamycin treatment increased the number of LC3B-positive autophagosomes and the co-localization of SNAP29 with autophagosomes (S3 Fig). We found that SNAP29 does not play a role in endothelial exocytosis (Fig 3). However, given the role of SNAP29 in maintaining various intracellular membrane trafficking steps, particularly autophagy which affects VWF processing [47], SNAP29 might be involved in regulating VWF degradation, possibly by regulating WPB-autophagosome or WPB-autophagosome-lysosome fusion, while SNAP23 preferentially regulates vesicle fusion with the plasma membrane. Our result is consistent with prior studies showing SNAP29 is primarily an intracellular SNARE [57], and does not appear to affect exocytosis [61, 67]

In summary, we have shown that human endothelial cells express SNAP23, SNAP23 interacts with other endothelial SNAREs, and SNAP23 plays a crucial role in endothelial exocytosis of VWF as a t-SNARE. This matches a model in which a three membered SNARE complex forms before endothelial exocytosis, consisting of a VAMP on the membrane of endothelial granules, along with STX4 and SNAP23 on the plasma membrane. Extending this model, other regulatory molecules such as synaptotagmin isoforms, Sec/Munc proteins, complexins, or STXBP5 could interact with these three SNARE members, further modulating exocytosis.

## Supporting Information

S1 Fig Minimal SNAP29 and SNAP47 protein expression in HUVEC. 20 μg protein from lysates of HL-60 cells or rat brain or HUVEC was fractionated and immunoblotted with antibodies to SNAP29, SNAP47, and GAPDH. Minimal levels of SNAP29 and SNAP47 are detected in HUVEC.
(TIF)

S2 Fig Subcellular localization of SNAP29 in endothelial cells. Confocal microscopy was used to define the location of SNAP29 in relation to VWF (A) and LC3B (B) in resting HUVEC. (A) Immunofluorescent staining of SNAP29 (red), VWF (green), and DNA (blue). Enlargement of boxed regions are shown in the lower panels. (objective 60× oil, upper panel scale bar = 40 μm, lower panel scale bar = 10 μm). (B) Immunofluorescent staining of SNAP29 (red), LC3B (green), and DNA (blue). Enlargement of boxed regions are shown in the lower panels. (objective 60× oil, upper panel scale bar = 40 μm, lower panel scale bar = 5 μm).
(TIF)

S3 Fig Subcellular localization of SNAP29, VWF, and LC3B upon induced autophagy in endothelial cells. HUVEC were starved (A) or treated with rapamycin (B) to induce autophagy. Confocal microscopy was used to define the location of SNAP29 (white) in relation to VWF (green) and LC3B (red) after autophagy induction. Enlargement of boxed regions are shown in the adjacent panels to the right. (objective 60× oil, left panel scale bar = 40 μm, enlarged panel scale bar = 10 μm).
(TIF)

S4 Fig SNAP23 co-localizes with endothelial exocytic machinery. HUVEC were treated with media or histamine 10 μM for 30 min, fixed, permeabilized, and stained with antibodies to SNAP23, STX4, VAMP3, and VAMP8. Cells were analyzed by confocal microscopy (objective 60× oil, scale bar = 40 μm).
(TIF)

S5 Fig Pearson correlation coefficient between SNAP23 and other SNAREs. Data from S4 Fig was quantified and the Pearson’s correlation coefficient was calculated (n = 4–7 ± S.D. NS = non-significant). SNAP23 is most co-localized with STX4, and less co-localized with VAMP3 or VAMP8.
(TIF)
