# Correction: SNAP23 Regulates Endothelial Exocytosis of von Willebrand Factor

**DOI:** 10.1371/journal.pone.0147167

**Published:** 2016-01-11

**Authors:** Qiuyu Martin Zhu, Munekazu Yamakuchi, Charles J. Lowenstein

The first author’s name is spelled incorrectly. The correct name is: Qiuyu Martin Zhu. The correct citation is: Zhu QM, Yamakuchi M, Lowenstein CJ (2015) SNAP23 Regulates Endothelial Exocytosis of von Willebrand Factor. PLoS ONE 10(8): e0118737. doi:10.1371/journal.pone.0118737
