# Supplementary figures and images for: SNAP23 Regulates Endothelial Exocytosis of von Willebrand Factor
Source: PLoS One. 2015 Aug 12;10(8):e0118737. doi: 10.1371/journal.pone.0118737 (PMC4534191; doi:10.1371/journal.pone.0118737)

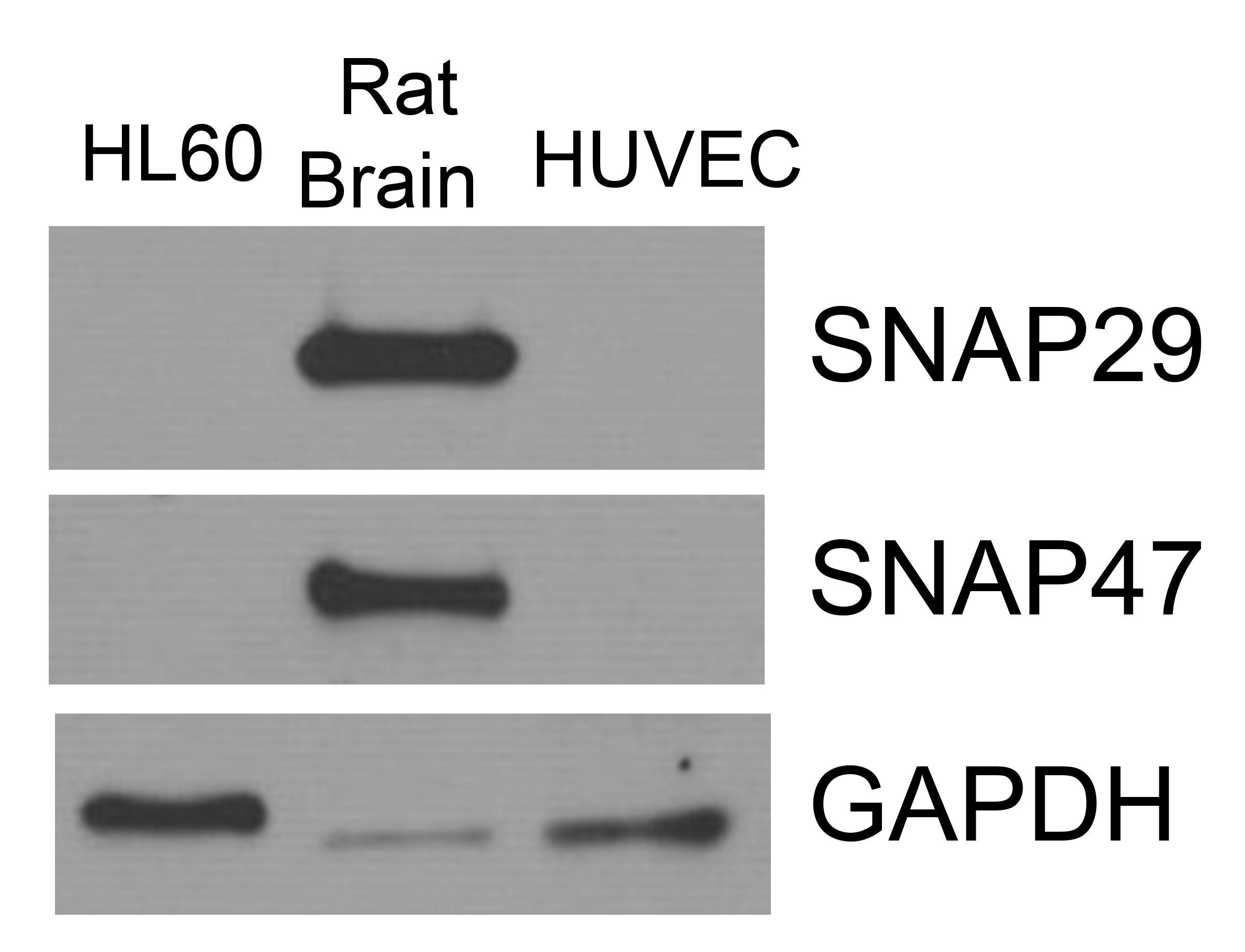

Supplement: S1 Fig — 20 μg protein from lysates of HL-60 cells or rat brain or HUVEC was fractionated and immunoblotted with antibodies to SNAP29, SNAP47, and GAPDH. Minimal levels of SNAP29 and SNAP47 are detected in HUVEC. (TIF) [file pone.0118737.s001.tif]

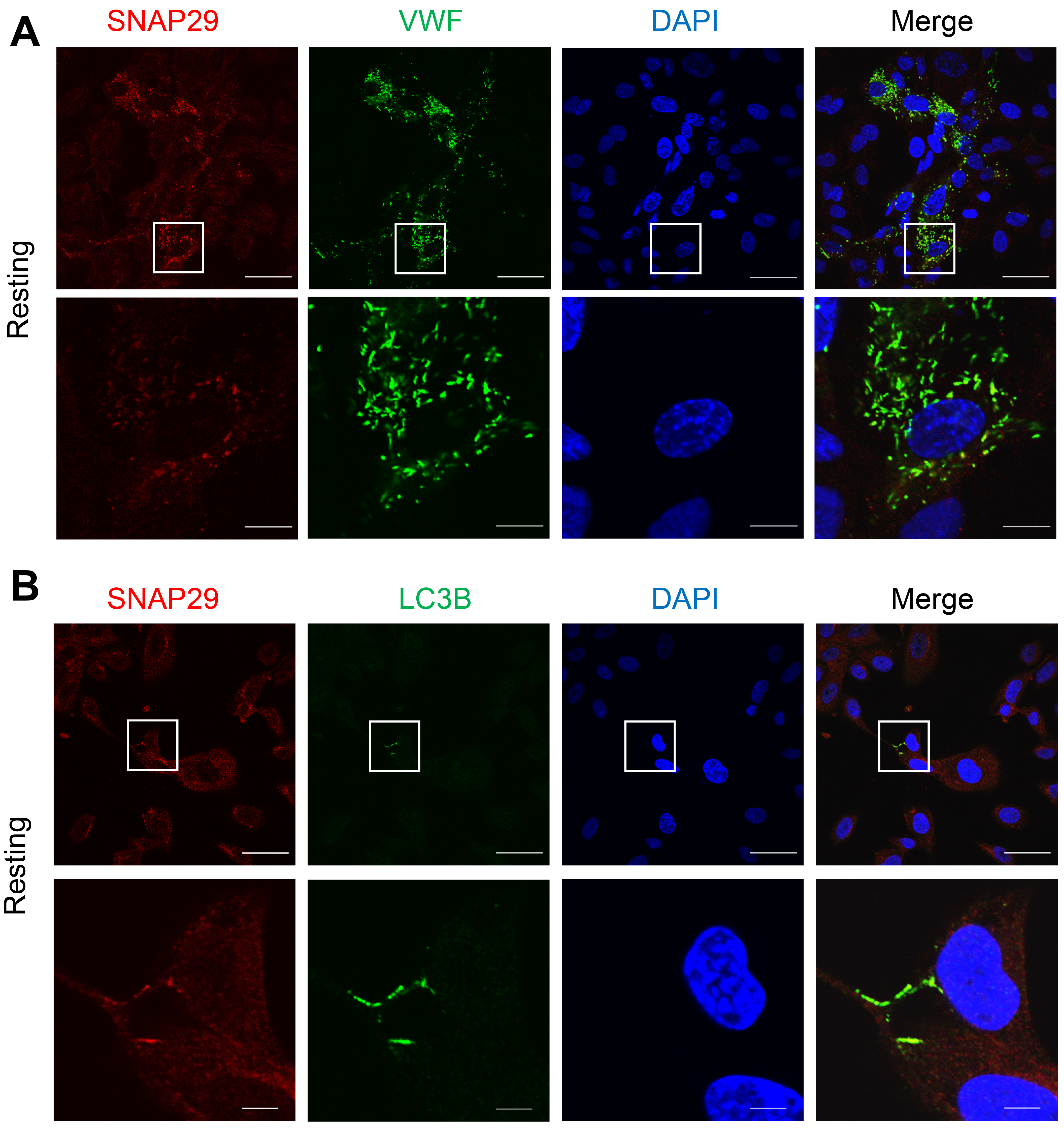

Supplement: S2 Fig — Confocal microscopy was used to define the location of SNAP29 in relation to VWF (A) and LC3B (B) in resting HUVEC. (A) Immunofluorescent staining of SNAP29 (red), VWF (green), and DNA (blue). Enlargement of boxed regions are shown in the lower panels. (objective 60× oil, upper panel scale bar = 40 μm, lower panel scale bar = 10 μm). (B) Immunofluorescent staining of SNAP29 (red), LC3B (green), and DNA (blue). Enlargement of boxed regions are shown in the lower panels. (objective 60× oil, upper panel scale bar = 40 μm, lower panel scale bar = 5 μm). (TIF) [file pone.0118737.s002.tif]

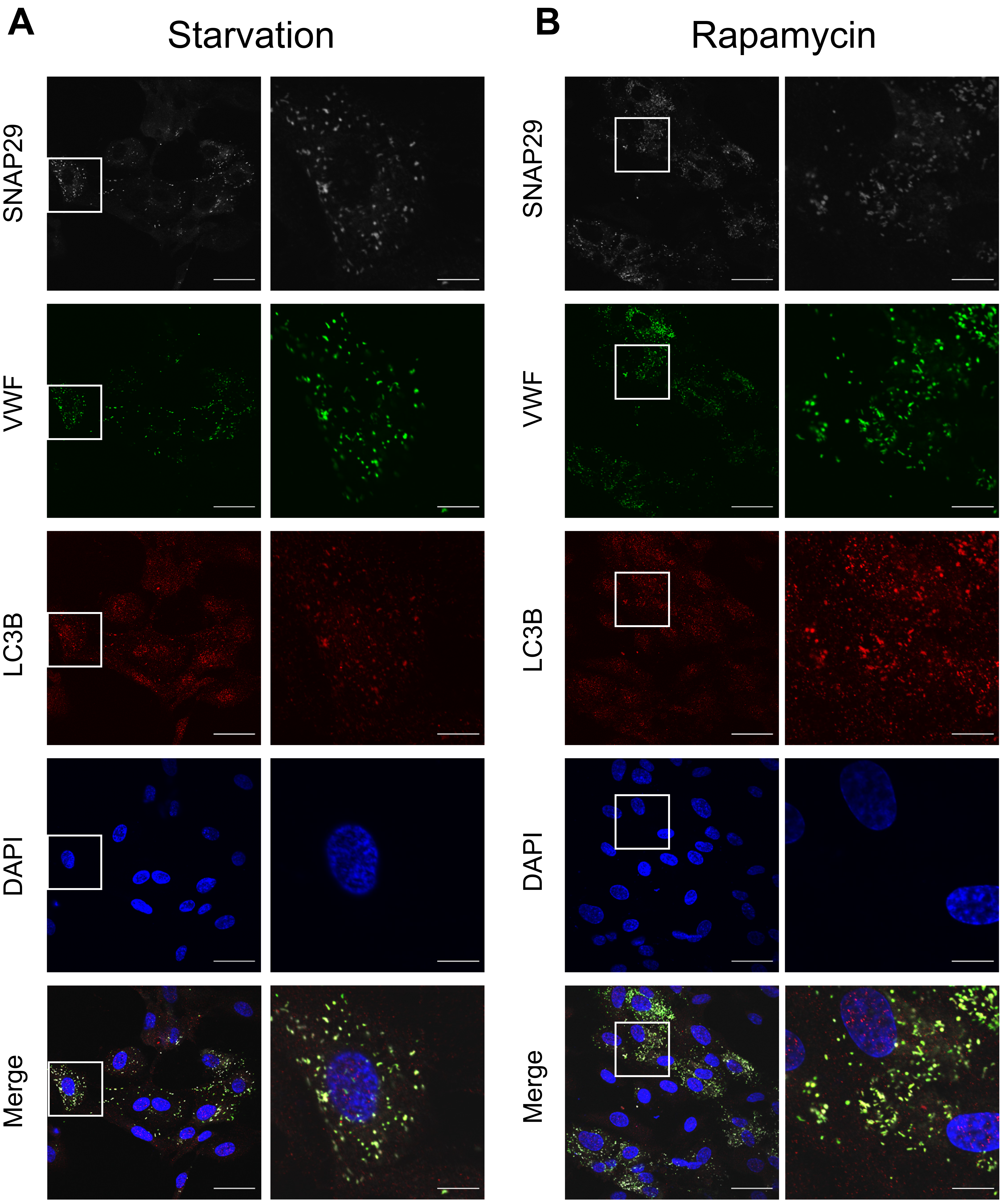

Supplement: S3 Fig — HUVEC were starved (A) or treated with rapamycin (B) to induce autophagy. Confocal microscopy was used to define the location of SNAP29 (white) in relation to VWF (green) and LC3B (red) after autophagy induction. Enlargement of boxed regions are shown in the adjacent panels to the right. (objective 60× oil, left panel scale bar = 40 μm, enlarged panel scale bar = 10 μm). (TIF) [file pone.0118737.s003.tif]

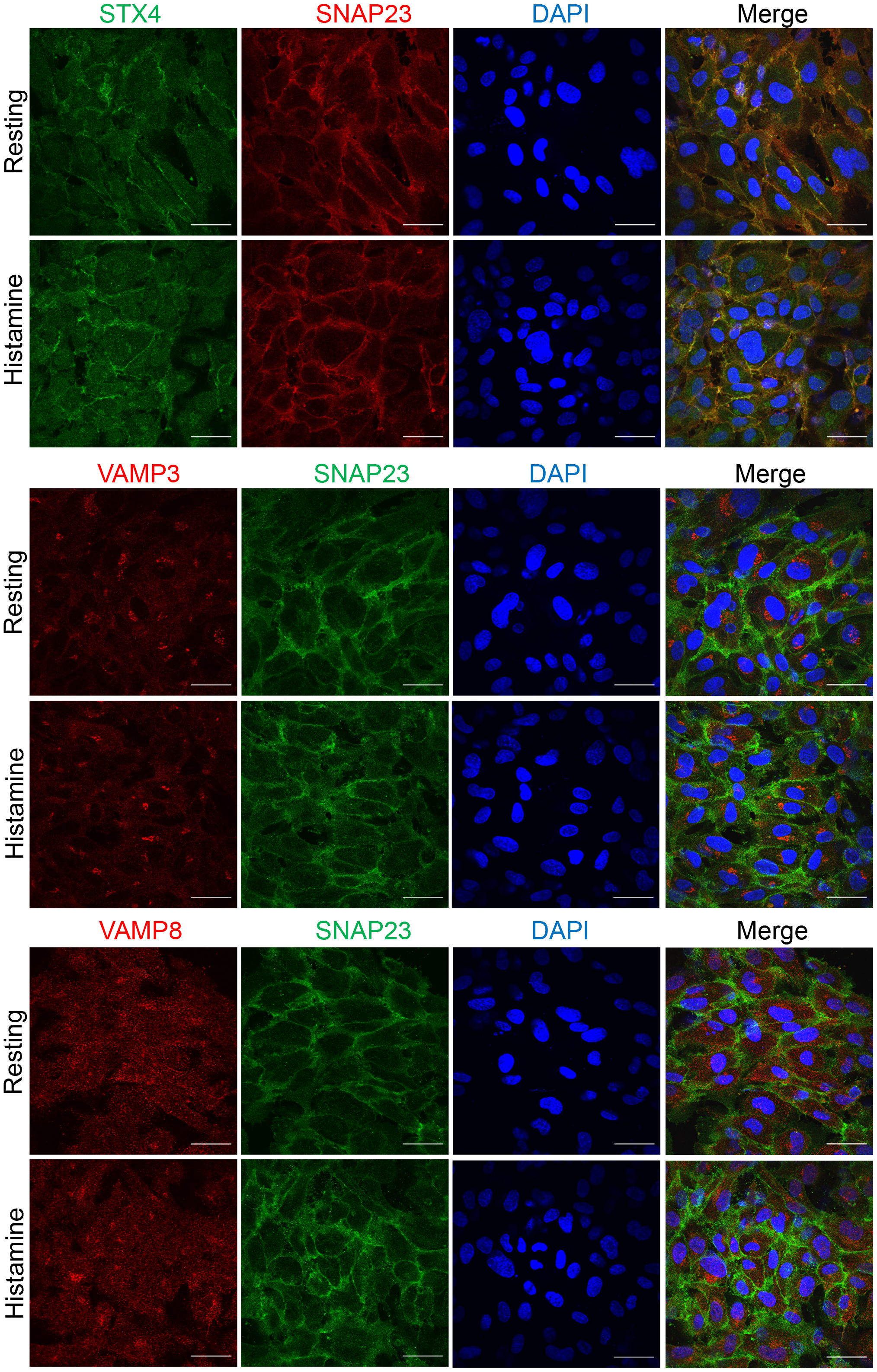

Supplement: S4 Fig — HUVEC were treated with media or histamine 10 μM for 30 min, fixed, permeabilized, and stained with antibodies to SNAP23, STX4, VAMP3, and VAMP8. Cells were analyzed by confocal microscopy (objective 60× oil, scale bar = 40 μm). (TIF) [file pone.0118737.s004.tif]

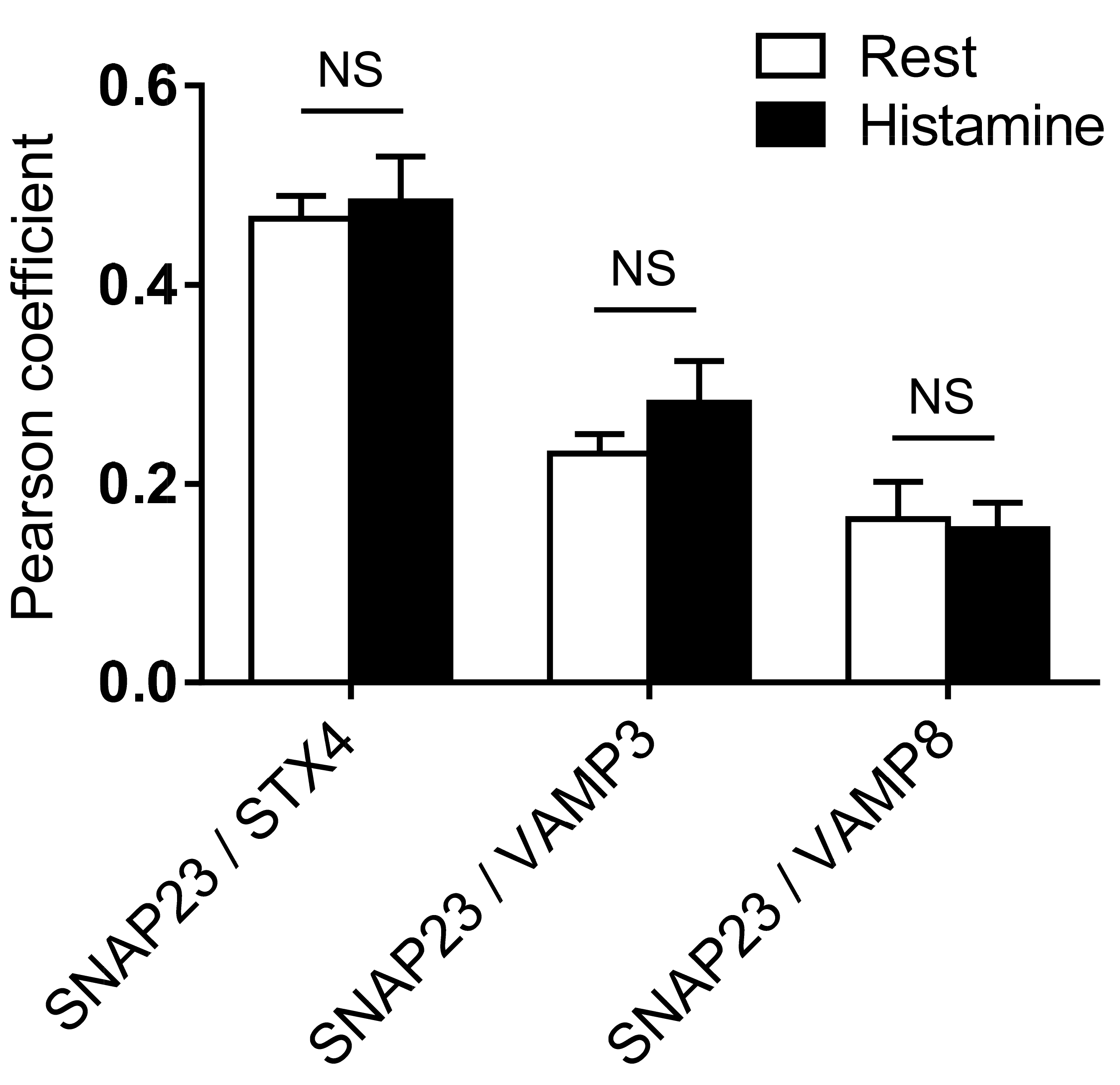

Supplement: S5 Fig — Data from S4 Fig was quantified and the Pearson’s correlation coefficient was calculated (n = 4–7 ± S.D. NS = non-significant). SNAP23 is most co-localized with STX4, and less co-localized with VAMP3 or VAMP8. (TIF) [file pone.0118737.s005.tif]
